# One-Enzyme RTX-PCR for the Detection of RNA Viruses from Multiple Virus Genera and Crop Plants

**DOI:** 10.3390/v14020298

**Published:** 2022-01-31

**Authors:** Hana Hoffmeisterová, Kateřina Kratochvílová, Noemi Čeřovská, Lucie Slavíková, Jakub Dušek, Karel Muller, Jan Fousek, Helena Plchová, Oldřich Navrátil, Jiban Kumar Kundu, Tomáš Moravec

**Affiliations:** 1 Laboratory of Virology-Centre for Plant Virus Research, Institute of Experimental Botany of the Czech Academy of Sciences, Rozvojová 263, 165 02 Prague, Czech Republic; hoffmeisterova@ueb.cas.cz (H.H.); kratochvilova@ueb.cas.cz (K.K.); cerovska@ueb.cas.cz (N.Č.); dusek@ueb.cas.cz (J.D.); fousek@ueb.cas.cz (J.F.); plchova@ueb.cas.cz (H.P.); navratil@ueb.cas.cz (O.N.); 2 Department of Experimental Plant Biology, Faculty of Science, Charles University in Prague, Albertov 6, 128 43 Prague, Czech Republic; 3 Plant Virus and Vector Interactions-Centre for Plant Virus Research, Crop Research Institute, Drnovská 507, 161 06 Prague, Czech Republic; slavikova@vurv.cz; 4 Department of Plant Protection, Czech University of Life Sciences, Kamýcká 129, 165 00 Prague, Czech Republic; 5 Laboratory of Hormonal Regulations in Plants, Institute of Experimental Botany of the Czech Academy of Sciences, Rozvojová 263, 165 02 Prague, Czech Republic; muller@ueb.cas.cz

**Keywords:** virus detection, Tobamovirus, Potyvirus, Polerovirus, Luteovirus, Potexvirus, Nepovirus, Tritimovirus, Foveavirus, Capillovirus, Trichovirus, RTX-PCR, one-step RT-PCR

## Abstract

Reverse transcription PCR (RT-PCR) is a popular method for detecting RNA viruses in plants. RT-PCR is usually performed in a classical two-step procedure: in the first step, cDNA is synthesized by reverse transcriptase (RT), followed by PCR amplification by a thermostable polymerase in a separate tube in the second step. However, one-step kits containing multiple enzymes optimized for RT and PCR amplification in a single tube can also be used. Here, we describe an RT-PCR single-enzyme assay based on an RTX DNA polymerase that has both RT and polymerase activities. The expression plasmid pET_RTX_(exo-) was transferred to various *E. coli* genotypes that either compensated for codon bias (Rosetta-gami 2) or contained additional chaperones to promote solubility (BL21 (DE3) with plasmids pKJE8 or pTf2). The RTX enzyme was then purified and used for the RT-PCR assay. Several purified plant viruses (TMV, PVX, and PVY) were used to determine the efficiency of the assay compared to a commercial one-step RT-PCR kit. The RT-PCR assay with the RTX enzyme was validated for the detection of viruses from different genera using both total RNA and crude sap from infected plants. The detection endpoint of RTX-PCR for purified TMV was estimated to be approximately 0.01 pg of the whole virus per 25 µL reaction, corresponding to 6 virus particles/µL. Interestingly, the endpoint for detection of TMV from crude sap was also 0.01 pg per reaction in simulated crude plant extracts. The longest RNA fragment that could be amplified in a one-tube arrangement was 2379 bp long. The longest DNA fragment that could be amplified during a 10s extension was 6899 bp long. In total, we were able to detect 13 viruses from 11 genera using RTX-PCR. For each virus, two to three specific fragments were amplified. The RT-PCR assay using the RTX enzyme described here is a very robust, inexpensive, rapid, easy to perform, and sensitive single-enzyme assay for the detection of plant viruses.

## 1. Introduction

Viral diseases are a major concern to sustainable agriculture worldwide, as they are ubiquitous and cause crop losses. In many cases, climate change is leading to an increase in disease incidence, host range, and pathogenicity [1,2,3]. Therefore, it is critical to monitor plant health and detect viral pathogens (pathogens as a whole) using low input, sufficiently sensitive and target-specific techniques. A wide range of virus diagnostic tools are available, including the observation of symptoms in indicator plants, electron microscopy, and methods based on the detection of viral proteins (e.g., serological methods) and viral nucleic acids (based on DNA amplification or RNA-seq). Techniques based on amplification of nucleic acid, especially those based on PCR (RT-PCR) or/and quantitative assay (RT-qPCR), have become standard tools for viral diagnostics over the years (for a review, see [4,5,6]).

One of the main concerns of PCR, especially the methods based on RT-PCR, is to perform the assay in one step, i.e., both reverse transcription and DNA polymerization in a single tube reaction, to avoid cross-contamination between samples. Another crucial aspect for RNA virus detection is the thermostability and reliability of the reverse transcriptase used for cDNA synthesis, especially in a one-step assay [7]. An elevated reaction temperature for cDNA synthesis is advantageous to overcome the strong secondary structures that are often present in RNA virus genomes; however, at the same time, most natural reverse transcriptases are not thermostable enzymes [7]. To overcome this problem, Andrew Ellington’s group used the strategy of in vitro evolution and developed a novel variant of the thermostable DNA polymerase KOD, originally found in the hyperthermophilic archaeon *Thermococcus kodakarensis*. The novel xenopolymerase/reverse transcriptase was named RTX [8]. The enzyme is stable at temperatures up to 98 °C and has proofreading activity unique to reverse transcriptase for both DNA and RNA templates [8]. Therefore, the RTX enzyme is well suited for the direct amplification of DNA from an RNA template in a single-enzyme reverse-transcription polymerase chain reaction (single-enzyme RTX-PCR), as well as from a DNA template. The traditional RTX-based one-step RT-PCR has been shown to be more effective than assays based on retroviral RT and thermostable DNA polymerase [9]. RTX has a high processivity for RNA templates, and fragments larger than 5 kb can be amplified during RT-PCR [8].

In this work, we describe the purification protocol for the RTX enzyme and its application for the detection of RNA plant viruses from different genera in diverse crop plants, including both monocotyledons and dicotyledons (both annual and perennial crops). The detection efficiency and endpoint detection limit of the one-step (one enzyme) RTX-PCR assay are also described and discussed. To our knowledge, this is the first report of an RTX-based assay for the detection of plant viruses.

## 2. Materials and Methods

### 2.1. Expression and Purification of Recombinant RTX

The expression plasmid pET_RTX_(exo-) was kindly provided by Andrew Ellington (Addgene Plasmid # 102786). The plasmid was transferred via the heat shock method to different *E. coli* genotypes [10]: BL21 (DE3); Rosetta-gami 2(DE3) (Novagen), carrying additional tRNA genes that compensate for the codon bias of *E. coli*; and BL21 (DE3) cells, carrying the plasmids pG-KJE8 and pG-Tf2 (Takara, Japan), which provide additional chaperones that may improve the soluble protein yield. Single colonies were used to inoculate 4 mL cultures of LB media containing the appropriate antibiotics and were grown overnight at 37 °C and 200 RPM. The saturated cultures were used to inoculate 200 mL of Dynamite media [11] containing the appropriate antibiotics and 0.05% AF204 antifoam (Sigma) in 1 L wide-mouth PET bottles. When the OD600 reached approximately 0.4, chaperone production was induced by adding l-arabinose (Biosynth, Sankt Gallen, Switzerland) at a final concentration of 0.1% (pG-Tf2) or by adding 0.1% arabinose and 5 ng/mL tetracycline (pG-Kje8). After an additional hour of incubation at 37 °C, all cultures were removed and placed on ice for 10 min to cool. Protein expression was induced by the addition of IPTG (Biosynth, Sankt Gallen, Switzerland) at a final concentration of 0.5 mM. The cultures were shaken overnight at 200 RPM and maintained at 22 °C. Finally, the cells were harvested by centrifugation at 4000× *g* for 20 min in 50 mL Falcon tubes. The pellets were weighed and frozen for further processing.

The enzyme was purified using a combination of heat treatment, DNA precipitation, and DNase treatment and ethanol precipitation, similar to the previously described method used for *Taq* polymerase [12]. Briefly, the cell pellets were resuspended in buffer A (50 mM Tris-HCl, pH 7.9; 50 mM dextrose, 1 mM EDTA, 1 mM PMSF, 10 mL per 1 g). Cell lysis was supported by the addition of 1 volume of lysis buffer (10 mM Tris-HCl, pH 7.9; 50 mM KCl, 1 mM EDTA, 1 mM DTT, 1 mM PMSF, 0.5% Tween 20, 0.5% Nonidet P40), 1:100 of the volume of CellLytic B cell lysis reagent (Sigma. St. Louis, MO, USA), 0.3 mg/mL hen’s egg lysozyme (Sigma), and 3 freeze–thaw cycles. The lysates were heated in a water bath at 75 °C for 20 min and the precipitated *E. coli* proteins were removed by centrifugation (20 min at 15,000× *g*). Streptomycin sulphate (Sigma) was added slowly to a total concentration of 0.5 g/100 mL lysate. DNase (Thermo Scientific, Waltham, MA, USA) and 10 mM MnCl2 were added to the supernatant and the reaction was incubated at 37 °C for 30 min. The DNase was deactivated by heat treatment (75 °C for 20 min) and removed by centrifugation (20 min at 15,000× *g*). Finally, RTX polymerase was precipitated by the addition of ethanol to a final concentration of 50%. The tubes were incubated on ice for 1 h, and the precipitated protein was resuspended in 10 mL of storage buffer (50 mM Tris-HCl, pH 7.9, 50 mM KCl, 0.1 mM EDTA, 1 mM DTT, 0.1% Nonidet P40, 0.1% Tween 20, 50% glycerol).

### 2.2. Virus Sources

Three purified viruses—Tobacco mosaic virus (TMV, U1 strain, genus Tobamovirus, accession number NC_001367.1), Potato virus X (PVX, genus Potexvirus, accession number AY297843.1), and Potato virus Y (PVY, NTN strain, genus Potyvirus, accession number AY166866.1)—were maintained in the Laboratory of Virology at the Institute of Experimental Botany (IEB-CAS) in Prague, Czech Republic. Other viruses, namely Tobacco ringspot virus (TRSV, genus Nepovirus, accession number KP144325), Turnip mosaic virus (TuMV, genus Potyvirus, accession number submitted to NCBI), Turnip yellows virus (TuYV, genus Polerovirus, accession number submitted to NCBI), Barley yellow dwarf virus (BYDV-PAV, genus Luteovirus, accession number FJ645745), Wheat streak mosaic virus (WSMV, genus Tritimovirus, accession number FJ216408), Apple stem pitting virus (ASPV, genus Foveavirus, accession number FJ970958), Apple stem grooving virus (ASGV, genus Capillovirus, accession number FJ952161), Plum pox virus (PPV, genus Potyvirus, accession number FJ842716), Apple chlorotic leaf spot virus (ACLSV, genus Trichovirus, accession number FJ952168), and Prune dwarf virus (PDV, genus Ilarvirus, accession number FJ842698I) were obtained from the microorganisms collection of the Crop Research Institute, Prague, Czech Republic.

### 2.3. Sample Preparation for Virus Detection (RNA Isolation, Virus Purification, and Crude Sap Preparation)

#### 2.3.1. Virus Purification

Three viruses (TMV, PVX, and PVY) were purified from the infected leaves of *Nicotiana benthamiana* according to the procedures outlined by Bruening et al. [13] (TMV) and Čeřovská et al. [14] (PVX and PVY). The plants were grown under LED illumination with 16 h of daylight, essentially as described by Janda et al. [15]. The concentrations of the purified viruses were estimated using a NanoDrop (Thermo Scientific Waltham, MA, USA) spectrophotometer and calculated using the corresponding molar extinction coefficients at A260 [16]. The concentrations of purified TMV, PVX, and PVY viruses were adjusted to 10 ng/µL in PBS and then serially diluted from 10^−1^ ng/µL to 10^−10^ ng/µL in PBS.

#### 2.3.2. RNA Isolation

Total RNA was extracted using TRIzol reagent (Thermo Scientific, Waltham, MA, USA) according to the manufacturer’s instructions. One millilitre of TRIzol reagent was added to 100 mg of homogenized plant tissue and mixed by pipetting. Then, 200 µL of chloroform was added to the tube, mixed by shaking, and incubated for 2–3 min at room temperature. The samples were centrifuged at 12,000× *g* for 15 min at 4 °C to separate the supernatant. After centrifugation, the upper aqueous phase was transferred to a fresh tube and 500 µL of isopropanol was added and incubated for 10 min at 4 °C to precipitate the RNA. After another centrifugation for 10 min at 12,000× *g* at 4 °C, the supernatant was discarded and the RNA pellet was resuspended in 1 mL of 75% ethanol by brief vortexing to remove the salts and organic residues. The tube was then centrifuged at 7500× *g* for 5 min at 4 °C, the supernatant was discarded, and the RNA pellet was dried for 10–15 min. The pellet was then re-suspended in 50 µL of RNase-free water by incubation at 55 °C for 10 min.

The concentration and purity of the isolated RNA was then measured spectrophotometrically (NanoDrop 2000; Thermo Scientific, Waltham, MA, USA).

#### 2.3.3. Crude Sap Preparation

Two types of sample preparation were performed.

First, the purified viruses (TMV, PVX, and PVY) were mixed with freshly harvested virus-free leaves of *Nicotiana benthamiana* that were crushed in crude extraction buffer (1:40 (*w/v*); 0.5 M Tris HCl pH 8.3 containing 0.15 M NaCl, 0.05% Tween 20, 2% PVP 40, 1% PEG 6000, and 0.003 M NaN3). The crude plant sap in the extraction buffer was divided into four aliquots and the purified TMV, PVX, and PVY were each added to an aliquot at a final concentration of 1 ng/µL, while the remaining aliquot served as a negative control. All aliquots were divided into two batches, and one batch was centrifuged at 10,000× *g* for 10 min to remove plant cell debris, while the second batch was left unprocessed. We then performed single-enzyme RTX-PCR analysis with the appropriate primers, using purified virus in PBS at a final concentration of 1 ng/µL as a positive control. The samples with purified TMV were also processed using a commercial one-step RT-PCR kit for comparison.

Second, we used the crude sap extracted from TMV-infected *N. benthamiana*. One hundred milligrams of leaf tissue were homogenized in a 2 mL tube with ceramic grinding beads using a MPBio FastPrep-24 homogenizer. The tubes were pretreated in liquid nitrogen. Alternatively, larger samples were homogenized in a pre-cooled mortar with a pestle. The homogenized plant materials were then suspended in 800 µL of the crude extraction buffer mentioned above. The suspensions were then centrifuged at 10,000× *g* for 10 min and the supernatants were serially diluted from 10^−1^ to 10^−5^ with the healthy crude extracts for each virus-infected sample. A 1 µL suspension of each dilution was used as a template for the one-enzyme RTX-PCR assay and for the one-step RT-PCR kit as a comparison.

### 2.4. One-Step RT-PCR with RTX and Commercial Qiagen One-Step Kit

#### 2.4.1. One-Enzyme RTX-PCR

One-enzyme RT-PCR with RTX was performed as follows: an RTX reaction mixture containing 2.5 µL of the RT buffer (250 mM Tris-HCl, 375 mM KCl, 15 mM MaCl_2_, and 100 mM DTT pH 8.3; the pH of the RT buffer is critical for a successful RTX-PCR reaction and should be measured before adding DTT), 1 µL of dNTP mixture (10 mM/L dNTPs), 0.3 µL of RTX enzyme, 2 µL of 100 mM (NH_4_)_2_SO_4_, 2 µL of upstream and downstream primer mixtures (100 mM; see Supplementary Table S1 [17,18,19,20,21,22,23,24,25,26,27,28,29,30,31,32,33,34,35,36,37,38]), 1000 ng/µL of total plant RNA (or purified viral RNA or crude sap) was prepared. The mixture was adjusted to 25 µL with RNase-free water.

The reaction in the classical (basic) setup was performed in a thermocycler (Bio-Rad, Hercules, CA, USA) as follows: an RT step at 68 °C for 30 min and then 33 cycles of 98 °C for 10 s (denaturation), 55–60 °C (based on the primer pair, see Supplementary Table S1) for 20 s (annealing), and 72 °C for 10–20 s (extension, based on the length of the amplified fragment). After the last cycle, a final extension step was added at 72 °C for 10 min.

For the quantitative RT-qPCR setup, the reaction conditions were as follows: 2 µL 5× buffer (300 mM Tris-HCl, 12.5 mM (NH_4_)_2_SO_4_, 50 mM KCl, 10 mM MgSO_4_, 2.5 mM betaine, pH 8.4), 0.4 µL 10 mM dNTP mixture, 0.5 µL 100 mM DTT, 0.25 µL EvaGreen dye (Biotium, Labmark, Czech Republic), 0.5 µL of each 10 mM primer, 30 ng of RTX exo-, and 2.5 µL of template (10 ng/µL–0.1 pg/µL TMV), in a total volume of 10 µL. The reactions were performed using a Roche LightCycler 480 II.

#### 2.4.2. One-Step RT-PCR with the Kit

One-step RT-PCR was performed using a one-step RT-PCR kit (Qiagen) as follows: A one-step mixture for RT-PCR containing 5 µL of 5× Qiagen one-step buffer RT-PCR, 1 µL of dNTP mixture (10 mmol/l dNTPs), 1 µL of Qiagen one-step enzyme mixture RT-PCR, 1 µL of Q solution, 2 µL of upstream and downstream primer mixtures (100 mM; see Supplementary Table S1), and 1000 ng of RNA (or purified viral RNA or crude juice) was prepared. The mixture was adjusted to 25 µL with RNase-free water. The reaction was performed in a thermocycler (Bio-Rad, Hercules, CA, USA) as follows: a RT step at 50 °C for 30 min and a first PCR activation step at 95 °C for 15 min, then 33 cycles of 94 °C for 30 s (denaturation), 55 °C for 45 s (annealing), and 72 °C for 80 s (extension). After the last cycle, a final extension step was added at 72 °C for 10 min.

## 3. Results and Discussion

### 3.1. RTX Enzyme Purification

Dr. Andrew Ellington’s group has described several protocols for the expression and purification of the RTX enzyme from *E. coli* [8,39]. We adapted and modified the protocols slightly to (a) optimize the yield of the soluble enzyme and (b) allow for the production of the purified enzyme in laboratories that lack the necessary equipment and expertise in protein chromatography.

**Figure 1:**
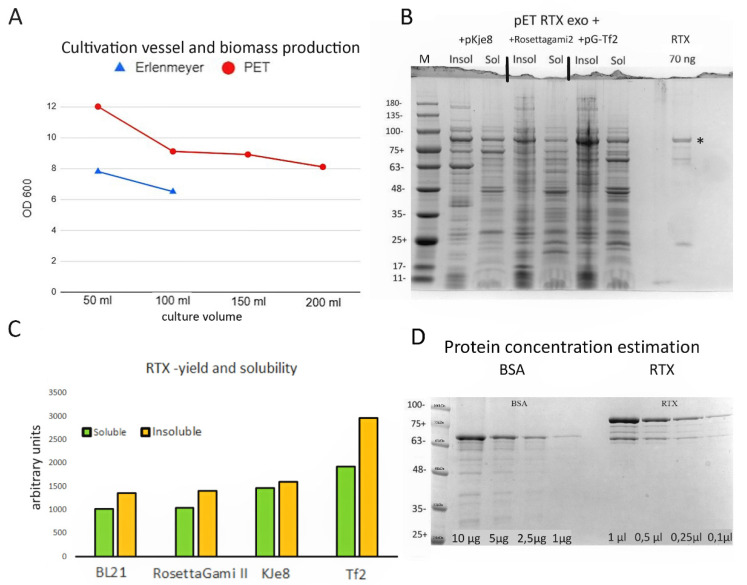
Characterization of the RTX exo- polymerase. (**A**) Biomass production measured as OD600 in either 250 mL Erlenmeyer flasks or 1 l PET flasks. ODs were measured 18 h after induction at saturation. (**B**) Coomassie blue-stained SDS-PAGE gel showing RTX expression and soluble/insoluble fraction ratios for BL -21 pKJe8, Rosetta-gami 2, and pG-Tf2 cells. For comparison, 70 ng of purified RTX enzyme was loaded into the last lane. The calculated molecular weight of the RTX enzyme was 90 kDa. The band corresponding to the full-size RTX enzyme is indicated by an asterisk. (**C**) Densitometric analysis of total RTX expression and partitioning between soluble and insoluble fractions. (**D**) Estimation of RTX enzyme concentration by densitometry. Known concentrations of BSA were used to generate a calibration curve that was used to calculate the amount of RTX exo- in the concentrated enzyme stock solution.

To this end, we used rich Dynamite media [11], which supports bacterial growth at very high densities. The flat-bottomed glass Erlenmeyer flasks we previously used provide limited aeration of the bacterial culture, resulting in poor growth and a low yield of soluble RTX protein. We tested the suitability of alternative fermentation vessels—1 L PET disposable wide-mouth bottles. These bottles are approximately the same diameter as 250 mL Erlenmeyer flasks; however, the baffled bottom greatly improves aeration. In an initial experiment, we compared the total bacterial biomass yield in glass Erlenmeyer flasks with the PET plastic bottles. Five millilitres of a saturated overnight culture of BL 21 cells with pET RTX exo+ was used to inoculate 1 l of Dynamite medium. The medium was then divided between 250 mL Erlenmeyer flasks (50 and 100 mL cultures) and PET bottles (50, 100, 150, and 200 mL cultures). After 3 h at 37 °C, the cultures were shifted to 21 °C to obtain higher amounts of active protein and induced with 0.5 mM IPTG. After 18 h, the ODs were measured. As shown in Figure 1A,C, biomass production per 1 mL was higher in the plastic flasks. Even a small culture volume of 50 mL in the glass flasks produced approximately the same biomass per 1 mL as the largest (200 mL) total volume in plastic bottles. The SDS-PAGE analysis showed a similar yield of soluble RTX protein in all samples. For further work, we used the plastic flasks containing 150 or 200 mL of culture medium.

Next, we hypothesised that the yield of soluble RTX protein could be improved either by co-expression with molecular chaperones or by enhancing the codon bias. The pET -RTX exo+ plasmid was transformed into BL21 DE3 cells, BL 21 Rosetta-gami 2 cells (Novagen), and BL21 cells with the chaperone-expressing plasmids pG-KJE8 (dnaJ/K and GroEs/GroEL chaperones, Takara) and pG-Tf2 (GroEs/GroEL, tig chaperones, Takara). After 1 h of the induction of chaperone expression with either arabinose alone or arabinose with tetracycline, the cultures were cooled to 21 °C and RTX expression was prompted by adding 0.5 mM IPTG. Samples collected after 18 h at 21 °C were subjected to SDS-PAGE and densitometric analysis. While the total yield and the percentage of soluble RTX enzyme in BL 21 and Rosetta-gami 2 cells were almost identical (43 and 42%, respectively), the cells with the pG-KJe8 plasmid showed a slight improvement in both the total yield and the percentage of soluble protein (48%). However, for routine expression, we chose BL -21 cells with the pG-Tf2 helper plasmid, which resulted in the highest total biomass yield. Although the ratio of soluble RTX protein was lowest in BL -21 pG-Tf2, this was compensated for by the improved overall protein expression. Based on the ChemiDoc MP imaging system software analysis (Biorad, Hercules, CA, USA), approximately 90% more soluble RTX protein was extracted from the same volume of culture than from normal BL21 DE3 cells (Figure 1B,C).

After finding the optimal growth conditions for RTX expression, we proceeded to protein purification. The enzyme was purified essentially as described in Chen et al. [12]. The RTX protein concentration in the final extract was estimated using densitometric analysis by SDS-PAGE against a BSA standard curve using the ChemiDoc MP imaging system software (Figure 1D). The final yield of the enzyme was estimated to be 8.5 mg/100 mL of medium. DNA polymerase activity was estimated by titration against DreamTaq and Phusion™ High-Fidelity DNA Polymerase (Thermo Scientific, Waltham, MA, USA) using a plasmid containing an infectious TMV clone and primers amplifying a 943 bp long amplicon (Supplementary Figure S1). The estimated activity was 40,000 U per 1 mg of purified enzyme. We diluted the enzyme to 125 µg/mL with the storage buffer. Aliquots of 1 mL were stored at −78 °C. Working aliquots were stored in a standard −20 °C freezer. We did not observe any reduction in activity after 1 year of storage in the freezer or after 2 days at room temperature. In this work, we used 40 ng of the enzyme in a standard RT-PCR reaction mixture of 25 µL. This protocol avoids both chromatography and dialysis and can be performed in any laboratory with a shaker incubator and centrifuge. Purification in one day yields enough enzyme to perform approximately 500,000 RT-PCR reactions from 200 mL of culture medium.

### 3.2. Efficiency of Virus Detection with Purified Viruses

**Figure 2:**
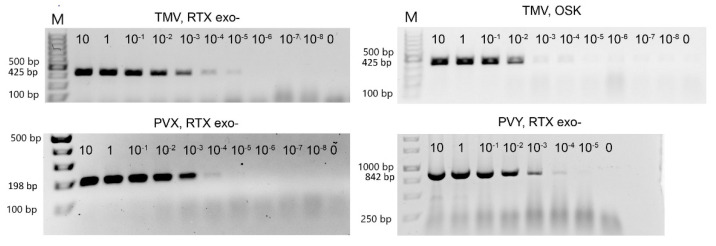
Determination of the detection limit of RTX-PCR by serial dilution. The marker in the left lane is the GeneRuler 100 bp Plus DNA Ladder from Thermo Scientific (Waltham, MA, USA), followed by a 10-fold serial dilution of TMV or PVX, respectively. PVY GeneRuler 1 kb DNA marker was used. The starting concentration was 10 ng of virus per 25 µL reaction. The upper right panel shows the dilution series of TMV amplified using the one-step RT-PCR kit (Qiagen). The size of the expected PCR amplicons is indicated on the left.

We used three purified viruses (PVX, PVY, and TMV) to determine the detection limits of both RTX and the commercial one-step RT-PCR kit. The reactions with the RTX enzyme provided sufficient sensitivity to reliably detect 1 pg of PVX and PVY or 0.01 pg of TMV per reaction (Figure 2). The detection limit for TMV was in the range of about six virus particles per 1 µL of reaction mixture, 70 virus particles per 1 µL for PVX, and approximately 400 virus particles per 1 µL for PVY. For TMV, the detection limits of RTX and the commercial one-step RT-PCR kit (Qiagen, Hilden, Germany) were compared. In this comparison, the sensitivity of the RTX reaction was slightly higher than the sensitivity of the one-step kit (approximately 1 pg per reaction vs. 0.01 pg in the RTX-based assay, see Figure 2). The sensitivity of the RTX-based assay is at least an order of magnitude higher than the sensitivities reported in the literature for similar protocols, such as standard RT-PCR [40] (1 pg for PVY), non-isotopic molecular hybridization [41] (5 pg), or one-step RT-PCR [42] (5 pg). Although we did not achieve such high sensitivity in the detection of PVX and PVY, these are still very low thresholds that are consistent with the values reported in the literature and are useful for most practical purposes. We hypothesise that the higher sensitivity of TMV detection compared to PVX or PVY may be the result of a complex relationship between the primers and template sequences and the viral particle shape and stability. The rod-shaped TMV particles are also less prone to aggregate formation; such aggregates might interfere with the generation of accurate dilution series.

**Figure 3:**
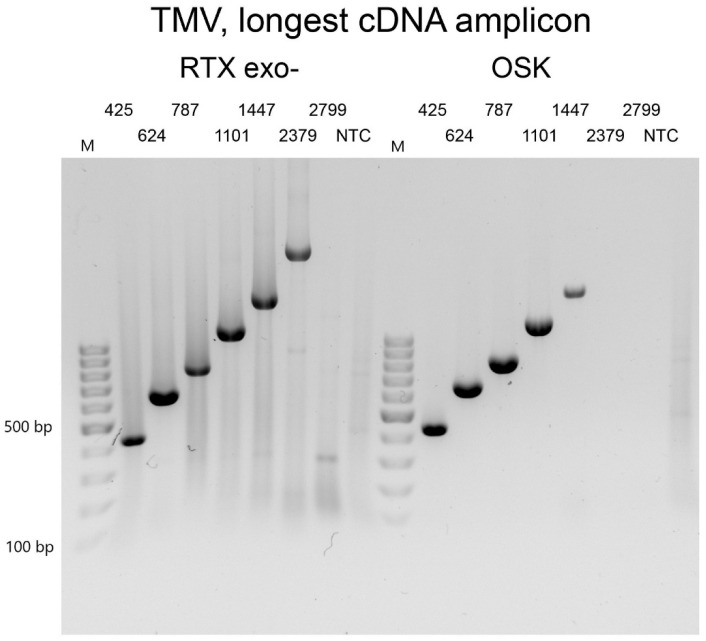
Determination of reverse transcription efficiency. Purified TMV particles were used as a template. All amplicons had the same reverse primer. The forward primer was progressively further positioned to generate amplicons of different lengths, from 425 bp to 2799 bp. The left panel shows reactions with RTXexo-, and the right panel shows reactions with the commercial one-step RT-PCR kit. NTC—non-template control. The marker is the GeneRuler 100 bp DNA Ladder from Thermo Scientific (Waltham, MA, USA).

Next, we sought to estimate the maximum length of RNA product that we could amplify with RTX polymerase in a single-tube reaction setup. For this experiment, we used 1 µL of a purified TMV preparation containing 10 ng/µL as the standard template for all reactions. All reactions contained an identical reverse primer that annealed to the 3’NTR region of the viral RNA and a unique sense primer to yield PCR amplicons of increasing size (425 bp, 624 bp, 787 bp, 1101 bp, 1447 bp, 2379 bp, 2779 bp, and 3304 bp). For the initial experiments, we used a standard 30 min reverse transcription. The maximum size of the amplicon was 1101 bp. After extending the RT step to 70 min, the length of the longest amplicon increased to 2379 bp. Further extension of the RT step beyond 70 min did not result in an increase in the size of the longest amplicon. Under comparable conditions, the longest PCR product amplified with the one-step RT-PCR kit was only 1447 bp (Figure 3). Therefore, the one-enzyme RTX-PCR kit would be preferable for the amplification of longer RNA fragments of viral genomes. This advantage might be related to the thermal stability of the reverse transcriptase, which might be required to overcome the strong secondary structures commonly found in viral genomes. Interestingly, Ellefson et al. [8] were able to amplify PCR products up to 5 kb in length from total RNA isolated from both prokaryotic and eukaryotic cells.

**Figure 4:**
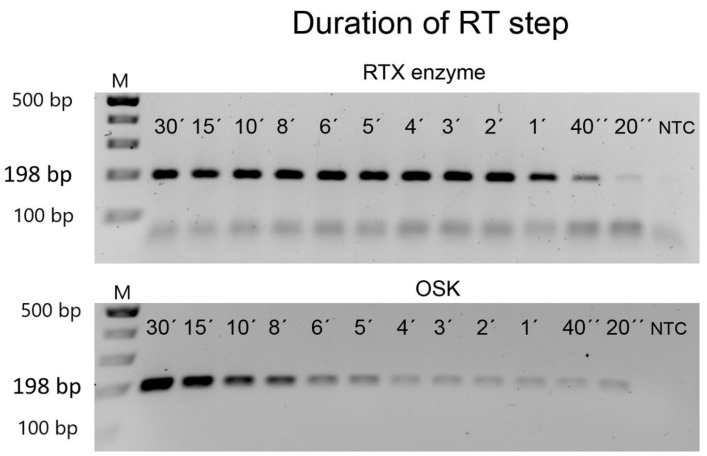
Estimation of the shortest reverse-transcription step. Purified PVX was used as a template to amplify a 198 bp specific amplicon. The standard reverse transcription step of 30 min was progressively shortened to only 20 s. NTC—non-template control. The marker is the GeneRuler 100 bp Plus DNA Ladder from Thermo Scientific. The size of the expected PCR amplicons is indicated on the left.

For some arrangements of RT-PCR, it is desirable to minimise the total reaction time. Therefore, we investigated if we could shorten the duration of the RT step for shorter amplicons. In this experiment, we selected a 198 bp amplicon from PVX and ran the RTX-PCR assay using a reverse transcription (RT) step of 30, 15, 10, 8, 6, 5, 4, 2, 1 min, 40 s, or 20 s, followed by the standard PCR steps of 33 cycles. As shown in Figure 4, even the shortest RT step of 20 s still amplified the target fragment; however, 40 s is sufficient to amplify a good quality RTX-PCR product. The performance of the commercial one-step RT-PCR kit was similar. Therefore, the required time for the detection of a virus by targeting short fragments can be significantly reduced by omitting or shortening the RT step in both assays (Figure 4).

### 3.3. Virus Detection Based on One-Enzyme RTX-PCR Using Crude Plant Sap

**Figure 5:**
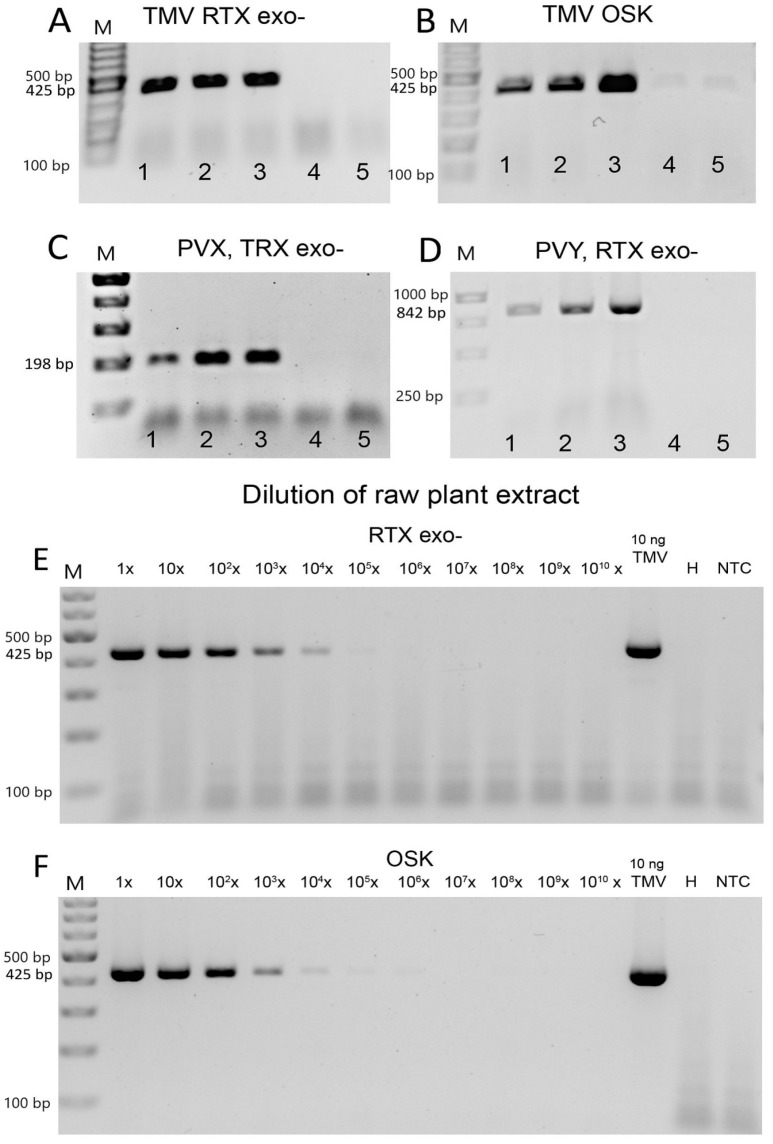
RT-PCR virus detection in crude extracts of *Nicotiana benthamiana*. The assays were performed with (**A**,**C**–**E**) RTX exo- polymerase or (**B**,**F**) with a commercial one-step RT-PCR kit. Three viruses were used as templates: (**A**,**B**,**E**,**F**) TMV; (**C**) PVX; or (**D**) PVY. (**A**–**D**) We attempted to establish the possibility of performing PCR with crude plant extracts. The numbers indicate different templates: 1—raw; unfiltered plant sap; 2—supernatant of raw extract; 3—purified virus in buffer; 4—healthy plant extract; 5—buffer only. (**E**,**F**) Estimation of virus detection limits in leaves of *N. benthamiana* naturally infected with TMV. NTC—non-template control. The marker in (**A**–**C**,**E**,**F**) is the GeneRuler 100 bp Plus DNA Ladder from Thermo Scientific, and in (**D**) the GeneRuler 1 kb DNA Ladder was used. The size of the expected PCR amplicons is indicated on the left.

The ability of the assay to work robustly with unpurified or only partially purified plant sap instead of purified total RNA samples would be highly desirable, especially for routine testing with a large number of samples. Therefore, we tested the RTX assay using raw plant sap, without any virus purification or RNA isolation, as a template. Alternatively, a commercial one-step RT-PCR kit (Qiagen) was used for comparison. In this experiment, we added 10 ng/µL of purified TMV, PVX, or PVY viruses to the raw plant sap of *Nicotiana benthamiana*. Both RTX and the commercial kit showed similar results for the untreated plant sap and its supernatant after 5 min at 14,000 rpm (Figure 5A–D).

Next, we determined the sensitivity of the assay in crude extracts. We used 10-fold dilution series of both extracts from *N. benthamiana* plants mechanically inoculated with TMV and a simulated dilution series with a known amount of TMV virus. In infected *N. benthamiana* sap, TMV was detected up to a dilution factor of 1:10,000 using both the single-enzyme RTX-PCR and the commercial one-step RT-PCR assay (Figure 5E,F). Based on the spiked dilution series, we concluded that the endpoint of TMV detection in raw plant juice could be estimated to be as low as 0.1 pg to 0.01 pg of virus per reaction, similar to the sensitivity to virus in buffer. These results indicated that the plant contaminants in the crude extracts of *N. benthamiana* did not significantly affect the ability of the RTX enzyme to amplify viral RNA and did not alter the detection limits. However, the combination of host materials and detected viruses with different virion properties may lead to different detection sensitivity. Such sensitivity provides sufficient room for pooling samples to further reduce costs. Thus, RT-PCR based on the RTX enzyme can serve as a robust and efficient tool for the routine detection and diagnosis of plant viruses. The ability of the RTX-based assay to reliably detect RNA viruses directly in crude pooled extracts, without the need for RNA isolation, immunocapture, a separate reverse transcription step, or other pre-processing steps significantly reduces both the time and cost of virus detection and also helps prevent potential cross-contamination between samples. Both the RTX enzyme and the commercial kit (Figure 5, Supplementary Figures S1 and S2) can be used with comparable sensitivity.

The detection of viruses from crude sap has been described elsewhere with similar efficiency, including the detection of ASPV and ASGV in apple leaves [43], Beet yellows virus (BYV) in sugar beet and *Tetragonia expansa* leaves [44], Lettuce necrotic yellows virus (LNYV) in lettuce leaves, Zucchini yellow mosaic virus (ZYMV) in squash leaves [45], and BYDV in oat leaves [46]. The crude sap-based one-step RT-PCR was also used to detect 16 virus species from 32 plant species in 15 families [42]. In all cases, a Tris-HCl buffer with various salts and additives was used to release the viral nucleic acid directly into the supernatant, either in the one-step RT-PCR or the traditional RT-PCR assay. However, in some cases, detection based on crude juice may have a rather low sensitivity [47], which is due to the presence of inhibitors [48] and depends on the plant species and tissues tested [42]. In contrast, the extraction buffer described here showed high sensitivity for the detection of TMV from crude sap. In recent years, several other virus detection assays have been described using raw plant sap as a template. The most popular of these is LAMP, which has been developed for a number of viruses [49,50]; however, the combination of primer and probe is still a challenge for successful virus detection. The lateral flow assay (LFA), based on the binding of virions with labelled antibodies or viral nucleic acids with labelled DNA or RNA probes [51,52], has also been described for virus detection from crude juice. The LFA has been used for multiplex virus detection [53] and for the quantification of viral titre [54]. Although LAMP and the LFA seem to be effective for virus detection without the need for RNA extraction or complex laboratory equipment, the single-enzyme method described here (RTX-PCR) is quite efficient for the detection of multiple viruses. Furthermore, the RTX-PCR method is based on conventional PCR with the added advantage of an easy-to-use single-enzyme system for the detection of RNA viruses.

### 3.4. Validation of the RTX-PCR Assay for the Detection of Virus Species from Different Genera and Crops

**Figure 6:**
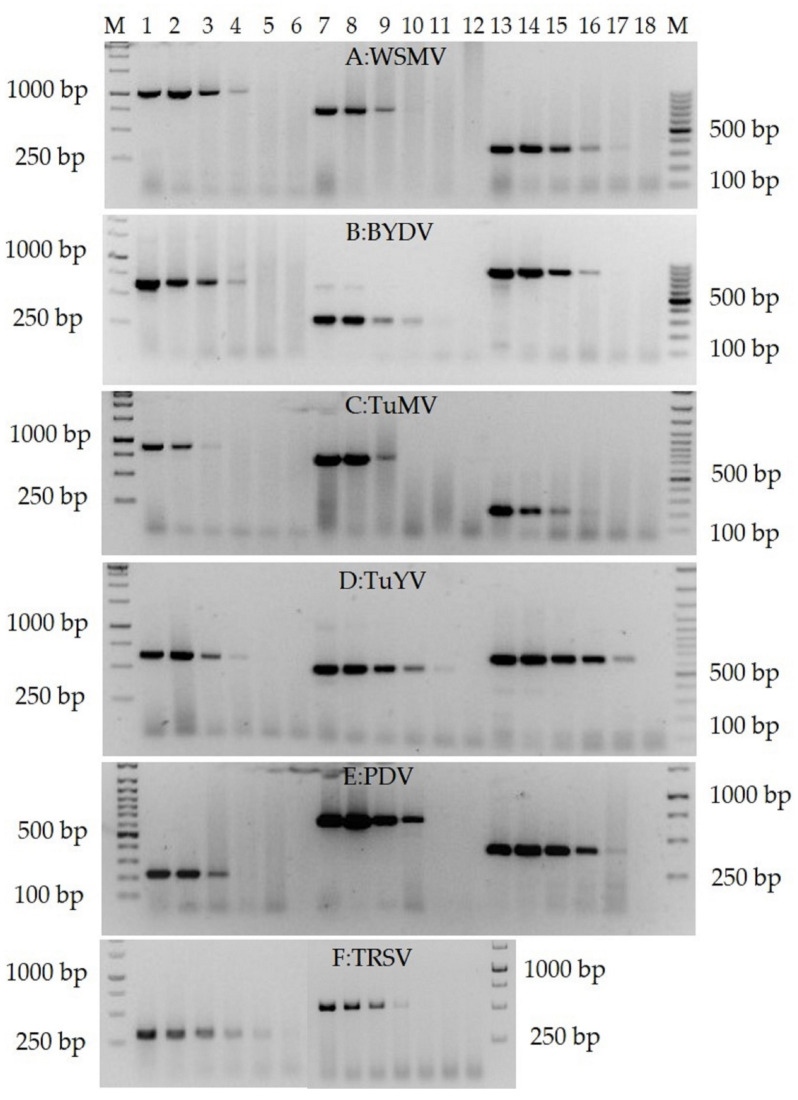
RTX-PCR pro detection of viruses (showing representatives of the detected viruses). (**A**) WSMV (total RNA of wheat leaves): Lane M—GeneRuler 1kb and 100 bp Plus DNA Ladder (Thermofisher Scientific, USA); lanes 1–6—primer pair WSMVcoatPRv/WSMVcoatPFv, 1014 bp; lanes 7–12—primer pair WSM8166/WSM8909, 743 bp; lanes 13–18—primer pair WSMVspeFv/WSMVspeRv, 354 bp. (**B**) BYDV (total RNA of barley leaves): lanes 1–6—primer pair BYcpF/BYcpR, 640 bp; lanes 7–12—primer pair BYDV-PVinterF/BYDV-Yan-Ra, 294 bp; lanes 13–18—primer pair BY5661R/BY4836F, 825 bp. (**C**) TuMV (total RNA of Chinese cabbage leaves): lanes 1–6—primer pair TuMV-full-CPF/TuMV-full-CPR, 896 bp; lanes 7–12—primer pair Nibfrg1F/Nibfrg1R, 737 bp; lanes 13–18—primer pair TuMV-F1qPCR/TuMV-F2qPCR, 208 bp. (**D**) TuYV (total RNA of oilseed rape leaves): lanes 1–6—primer pair TuYV-full-CPF/TuYVR-K2, 966 bp; lanes 7–12—primer pair luteoviruses-F/luteoviruses-R, 610 bp; lanes 13–18—primer pair TuYV-full-CPF/TuYV-full-CPR, 947 bp. (**E**) PDV (total RNA of plum leaves): lanes 1–6—primer pair PDVdpuF/PDVdpR, 220 bp; lanes 7–12—primer pair PDVcpF/PDVcpR, 687 bp; lanes 13–18—primer pair PDVrna2F/PDVrna2R, 418 bp. (**F**) TRSV (total RNA of tomato leaves): lanes 1–6—primer pair MF05-21-R/MF05-22-F, 320 bp; lanes 7–12—primer pair TRSV-Pr-F/TRSV-R, 523 bp. The serial dilutions of total RNA were 1:1 in lanes 1, 7, and 13 in (**A**–**E**) and lanes 1 and 7 in (**F**); 1:10 in lanes 2, 8, and 14 in (**A**–**E**) and lanes 2 and 8 in (**F**); 1:100 in lanes 3, 9, and 15 in (**A**–**E**) and lanes 3 and 9 in (**F**); 1:1000 in lanes 4, 10, and 16 in (**A**–**E**) and lanes 4 and 10 in (**F**); 1:10000 in lanes 5, 11, and 17 in (**A**–**E**) and lanes 5 and 11 in (**F**); 1:100000 in lanes 6, 12, and 18 in (**A**–**E**) and lanes 6 and 12 in (**F**).

We selected several RNA viruses from different virus genera that are the major pathogens of the main arable (cereals, oilseed rape, potatoes) and horticultural (pome and stone fruit trees, vegetables) crops (see Figure 6 and Supplementary Table S1). Different primer pairs were used to amplify specific fragments of the virus genomes from total RNA isolated from infected leaves (see Supplementary Table S1). The results indicated that the RT-PCR assay using the RTX enzyme could reliably amplify specific genome fragments of all tested viruses. As would be expected, the sensitivity of viral RNA detection was dependent on the different combinations of viruses and primer pairs. Based on the RNA template concentration and RNA dilution series, the estimated endpoint of detection ranged from 100 ng/µL to 0.1 ng/µL of total RNA in a reaction (Figure 6), depending on the virus–primer combinations, virus species, and plant material. A similar detection limit of 1 ng/µL total RNA was reported for four cucurbit-infecting viruses using a one-step RT-PCR method [55].

## 4. Conclusions

The RTX-PCR assay described here is based on a DNA polymerase from the hyperthermophilic archaeon *Thermococcus kodakarensis* that has both RT and polymerase activities and which was purified in our laboratory. The RTX expression plasmid is provided free of charge to academic and non-commercial entities and can be self-produced for scientific purposes. Currently, there is no commercial source for the RTX polymerase; however, the expression plasmid is available for academic laboratories from Addgene. For commercial, for-profit use, it may be necessary to obtain a licence from the University of Texas, Austin, USA.

Using the RTX enzyme, we developed an efficient and cost-effective RT-PCR assay for the detection of different species and genera of RNA viruses infecting various crops. The sensitivity of the single-enzyme RTX-PCR assay proved to be as high or higher than that of a commercially available one-step RT-PCR kit. The RTX-based assay also offered advantages in amplifying longer RNA templates and shortening or eliminating the RT step. On the other hand, the PCR products obtained with the commercial kit were generally cleaner, with no visible low molecular weight smear. While the presence of smears is not a significant problem in endpoint PCR, it could cause problems in qPCR assays (Supplementary Figure S3). We hypothesise that the presence of smearing could be related to impurities left in our RTX enzyme preparation. However, the smearing effect could also be the result of suboptimal buffer composition or reaction conditions. In conclusion, the RTX-PCR assay is easy to perform and is a time-saving procedure, as no initial RT step is required and the entire experiment can be completed within 60 min. In our laboratory, we have entirely replaced commercial enzymes and kits for virus detection with the RTX-based assay.

## Data Availability

The data sets are submitted together with the study.

## Supplementary Materials

The following are available online at https://www.mdpi.com/article/10.3390/v14020298/s1, Figure S1: Estimate of RTX polymerase processivity with DNA template; Figure S2: TMV detection limit in simulated plant extracts; Figure S3: Use of RTX for TMV quantification using RT-qPCR; Table S1: list of primers used in this work.
