# Supplementary material for: One-Enzyme RTX-PCR for the Detection of RNA Viruses from Multiple Virus Genera and Crop Plants
Source: Viruses. 2022 Jan 31;14(2):298. doi: 10.3390/v14020298 (PMC8924886; doi:10.3390/v14020298)
Supplement: Supplementary file 1 [file viruses-14-00298-s001.zip › viruses-1554559-supplementary.pdf]

viruses

MDPI

Supplementary Materials

One-Enzyme RTX-PCR for the Detection of RNA Viruses from Multiple Virus Genera and Crop Plants

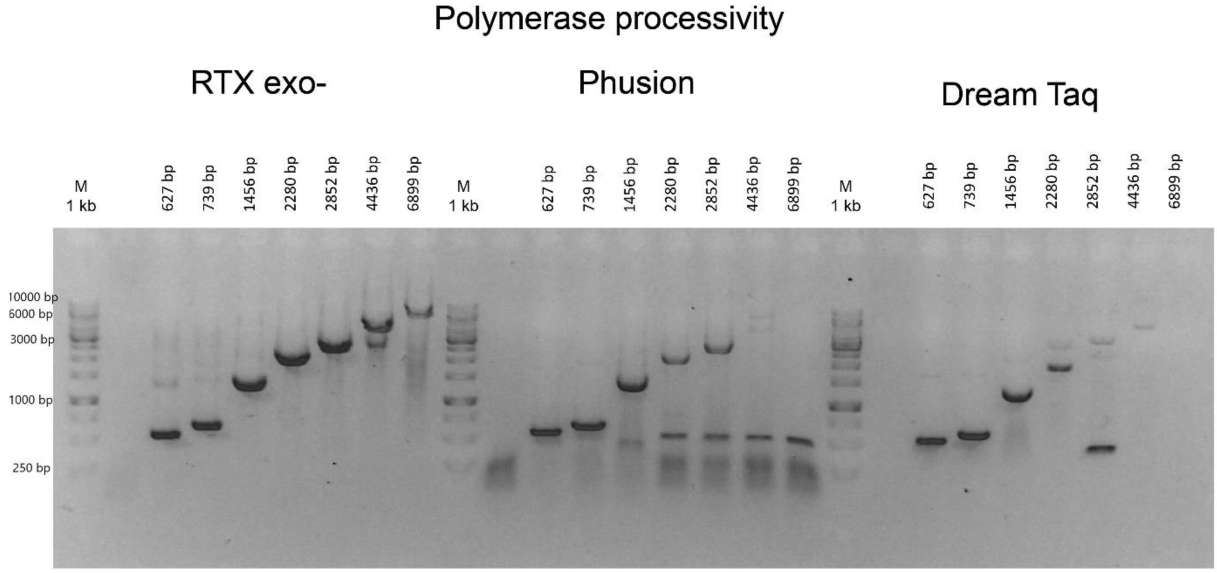

Figure S1. Estimate of processivity of thermostable polymerases. Universal domestication plasmid pUPD2 [56] with cloned inserts of various sizes (from 332 to 6604) were used as a template in PCR with universal sequencing primers pUPD F2/R2 [38]. 30 cycles of PCR program consisting of 30s denaturation at 94°C, 30s annealing at 55°C and short extension for 10s at 72°C were run with 3 different polymerases: RTX exo-, Phusion (ThermoScientific), and DreamTaq (ThermoScientific). Under these settings, the RTXexo- can be used to amplify products up to 5-6 kb in length. The marker is the GeneRuler 1 kb bp Plus DNA ladder from ThermoScientific.

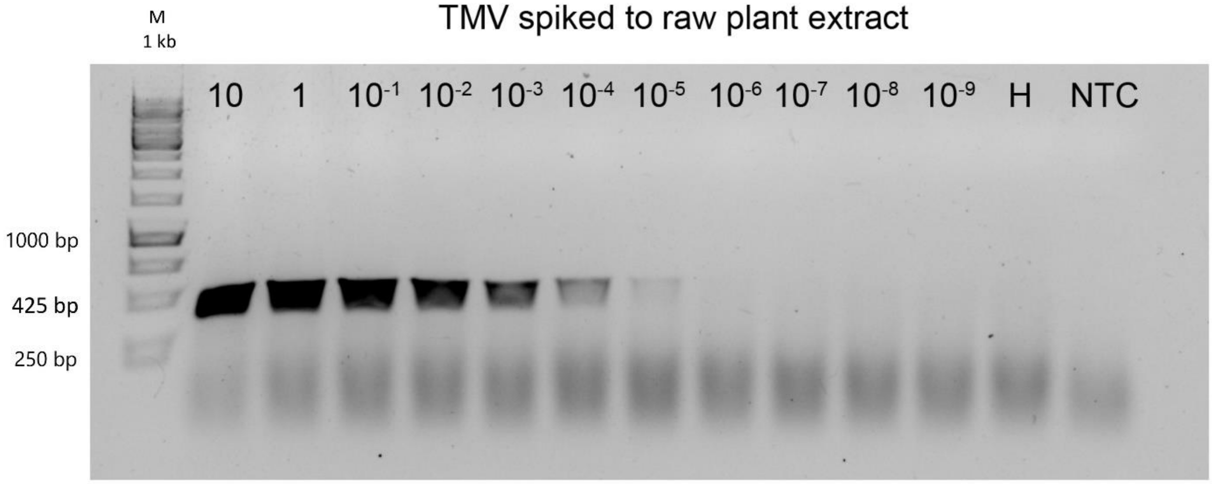

Figure S2. RT-PCR virus detection in crude extracts of Nicotiana benthamiana. Purified TMV was added to the extract of uninfected healthy plants to a final concentration of 10 ng/µl, and a 10-fold dilution series was prepared using extracts of healthy plants. 1 µl of the extracts was used as a template for RT-PCR using the RTX enzyme. Numbers indicate ng of pure TMV virus in each

reaction. H= extract from uninfected control plants, NTC = non-template control. The marker is the GeneRuler 1 kb bp Plus DNA ladder from ThermoScientific.

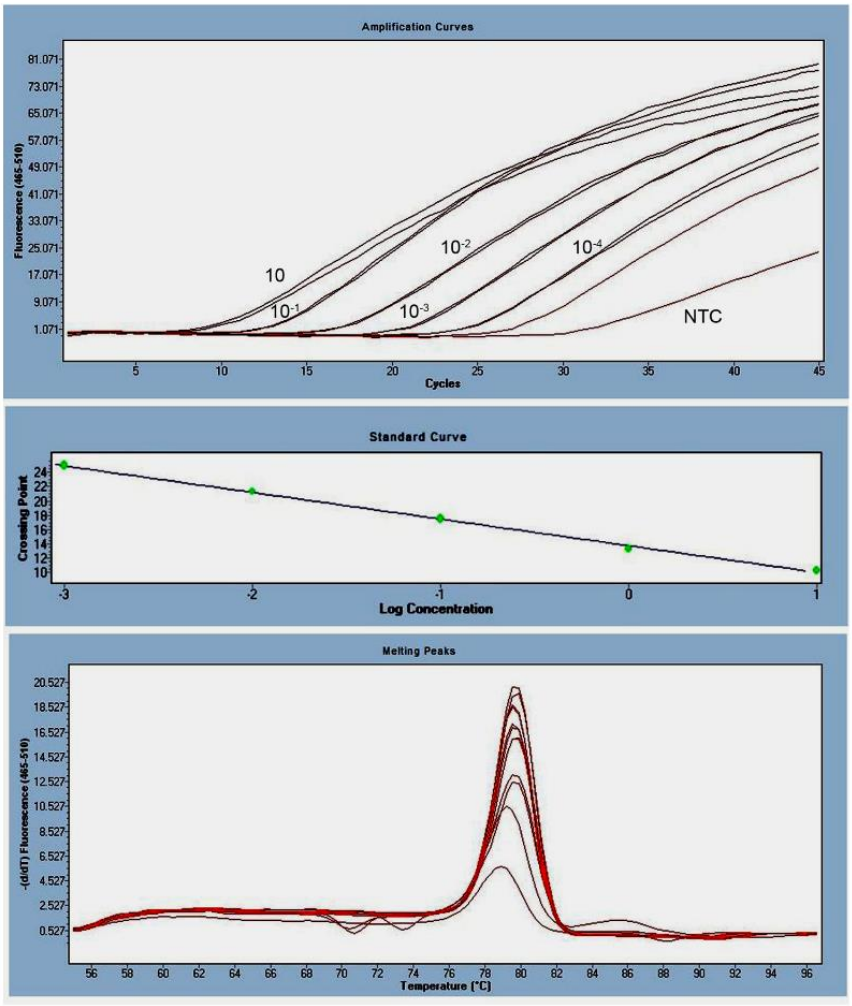

Figure S3. RT-qPCR reactions with the RTX exo- enzyme. Calibration curve constructed with the purified TMV preparation 10-fold serially diluted at 2.5 x 10ng to 2.5 x10^{-5} ng/reaction. Reactions were run in duplicates. The upper panel shows the real-time fluorescence measurements, the middle panel shows the standard calibration curve, and the lower panel shows the melting curve analysis of amplified fragments. .

Table S1. List of primers used in this work.

Virus	Genus	Primer Name	Primer sequence (5´-3´)	Nucleotide Position	PCR Fragment bp	Reference
Tobacco moasic virus (TMV)	*Tobamovirus*	TMV-MP-detAS	TGCAAGCCTGATCGACATAG	5507-5526		in this study
		TMV-MP-detS	TCTGTTTAGCCGGTTTGGTC	5102-5121	425	in this study
		Gb2-MP-F	*GCGCCGTCTCGCTCGA* ATGGCTCTAGTTGTTAAAGGAAAA*	4903-4926	624 + 16	in this study
		TMV-Rep-BsaI-S	GGAGTTCAGAAGATCTCTTTGTGATG	4740-4752	787	in this study
		TMV-LB	GAAACACTGTGATCATTGCTGC	4426-4447	1101	in this study
		TMV-rep-4080S	CCGTTGTTTAGTGAGCTTACTAGGC	4080-4104	1447	in this study
		TMV-rep-3148S	CTCTGCTTTCAAGAGGGTATTCAG	3148-3171	2379	in this study
		GB-TMV-3_4 F	*GCGCCGTCTCGCTCGAATG* CTTTCATGATGAATTTTGGGAAAAG*	2728-2752	2799 + 19	in this study
		TMV-rep-2223S	GCTGCGGTGTCGAATCTCGTCAAG	2223-2246	3304	in this study
		TMV-rep-1396S	TGGACAAATCTTTGTTACAATCCT	1396-1419	4131	in this study
		TMV qFor1	ATTAGACCCGCTAGTCACAGCAC	5939-2961		in this study
		TMV qRev1	TAGAGTAGACGACGCAACGGTG	6050-6071	131	in this study
Potato virus X (PVX)	*Potexvirus*	PVX-F3	AAGCTCCACAGGAAACACAG	1612-1631		in this study
		PVX-B3	GGTTGGGAGTGTGAGTTCTT	1790-1809	198	in this study
Potato virus Y (PVY)	*Potyvirus*	GB2-YCPF1	*GCGCCGTCTCGCTCGA* ATGGGAAATGACACAATCGATG*	8571-8589		in this study
		GB2-YCPR1	*GCGCCGTCTCGCTCAA* AGCTCACATGTTCTTGACTCCAAG*	9354-9374	804 + 19 + 19	in this study
		Mor1	AGGAGGAAGCACTAAGAAG	8591-8609	332	[17]
		Mor2	CAAACCATAAGCCCATTCATC	8903-8923		[17]
		MF05-21-R	CAATACGGTAAGTGCACACCCCG	5654-5632	320	[18]
		MF05-22-F	CAGGGGCGTGAGTGGGGGCTC	5334-5354		[18]
		TRSV-Pr-F	GGGGTGCTTACTGGCAAGG	3195-3213	521	[19]
		TRSV-R	ACTTGTGCCCAGGAGAGCTA	3716-3695		[20]
Turpin mosaic virus (TuMV)	*Potyvirus*	Nibfrg1F	GCACGGCAGTCTACGCACAAAC	7174-7195	679	in this study
		Nibfrg1R	CTGGTAACGACTCCAACAAGCG	7911-7890		in this study
		TuMV-full-CPF	GTGTTTATCACCAGGCAGGTG	8731-8751	896	[21]
		TuMV-full-CPR	GTCTACCAGCATACAACTTCAC	9627-9606		[21]

		TuMV-F1qPCR	GTTCAACGCGGAAGCAGTTT	9071-9090	208	in this study
		TuMV-R2qPCR	GCCTAAATGTGGGTTTGGCG	9279-9260		in this study
Turnip yellows virus (TuYV)	*Polerovirus*	luteoviruses-F	GCTCTAGAATTGTTAATGAITACGGTCG	generic primer	610	[22]
		luteoviruses-R	CACGCGTCIACCTATTTIGGITTITG	generic primer		[22]
		TuYV-full-CPR	CCGGGTTCCTCGTCTACCTA	4095-4076	947	in this study
		TuYV-full-CPF	CACGTACGCGAAATCGTTAATG	3148-3130	966	in this study
		TuYVR-K2	ATTGGTCCTCGGCAACGTCG	4114-4126		[23]
Barley yellow dwarf virus (BYDV)	*Luteovirus*	BYcpF	CCACTAGAGAGGTGGTGAAT	2840-2859	640	[24]
		BYcpR	CCGATGTTGAGGAGTCTACC	3479-3460		[24]
		BYDV-PVinterF	GTTGAGTTTAAGTCACACGC	3182 - 3201	294	[25]
		BYDV-Yan-Ra	TGTTGAGGAGTCTACCTATTTG	3475 - 3454		[25]
		BY5661R	TGCCGAACTGCTCTTTCGAGTG	5661-5640	825	in this study
		BY4836F	ATCCTGGGAAACAGGCAGAAC	4836-4856		in this study
Wheat streak mosaic virus (WSMV)	*Tritimovirus*	WSMVcoatPRv	GAAACTGTGCGTGTTCTCCC	9197-9178	1014	[26]
		WSMVcoatPFv	GCGTGTACGAATCGAGTGAG	8161-8181		[26]
		WSM8166	GAGAGCAATACTGCGTGTACG	8166-8186	743	[27]
		WSM8909	GCATAATGGCTCGAAGTGATG	8909-8929		[27]
		WSMVspeFv	GCCTCGACA CGGGAGCTA	8397-8417	354	[28]
		WSMVspeRv	ACC CATCCAGGAAGCAAGG	8754-8736		[28]
Apple stem pitting virus (ASPV)	*Foveavirus*	ASPV-A	ATAGCCGCCCCGGTTAGGTT	9237–9256	264	[29]
		ASPV-C	CTCTTGAACCAGCTGATGGC	8993–9012		[29]
		ASPV forward	CWAAYCCWTTTGAAACTGG	8312-8220	839	[30]
		ASPV reverse	GCTTGCGTCCAAYYTTTC	9151-9134		[30]
		ASPV sense	ATGTCTGGAACCTCATGCTGCAA	8869-8895	370	[31]
		ASPV antisense	TTGGGATCAACTTTACTAAAAAGCATAA	9211-9238		[31]
Apple stem grooving virus (ASGV)	*Capillovirus*	AGUV-2	GGAATTTCACACGACTCCTAACCCTCC	6345-6371	499	[32]
		AGUV-U	CCCGCTGTTGGATTTGATACACCTC	5873-5897		[32]
		ASGV forward	GTTTGGAAGACGTGCTTC	5644-5661	819	[30]
		ASGV reverse	ACACTAACCCGGAAATGC	6446-6463		[30]
		ASGV sense	GTTTGGAAGACGTGCTTC	6039-6064	272	[31]
		ASGV antisense	ACAACTAACCCGGAAATGC	6286-6311		[31]
Plum pox virus (PPV)	*Potyvirus*	PPV-F3	GGAATGTGGGTGATGATGG	9 153–9 171	345	[33]
		PPV-RR	CTCTTCTTGTGTTCCGACGTTTC	9 475–9 497		[33]
		mM3	CATTTCCATAAACTCCAAAAGAC	8 786–8 808	605	[34]
		mD5	TATGTCACATAAAGGCGTTCTC	8 207–8 228		[34]

		PPV-RR	CTCTTCTTGTGTTCCGACGTTTC	9 475–9 497	965	[33]
		RecJF	AATGATATTGATGATAGCCTTGAC	8532-8556		[35]
Apple chlorotic leaf spot virus (ACLSV)	*Trichovirus*	8RACLS	GCCTACAAATTAGGTGAGAGGCTC	5 851–5 832	290	[36]
		5FACLS	TTCCAATGGATCATGAGGTC	5564–5 585		[36]
		ACLSVFrII	CAAGAGAATTTCAGTTTGCTCG	6745-6766	791	[30]
		ACLSV antisense	AAGTCTACAGGCTATTTATTATAAGTCTAA	7507-7536		[31]
		ACLSV sense	TTCATGGAAAGACAGGGGCAA	6860-6880	647	[31]
		ACLSV antisense	AAGTCTACAGGCTATTTATTATAAGTCTAA	7507-7536		[31]
Prune dwarf virus (PDV)	*Ilarvirus*	PDVdpuF	CCGAGTGGATGCTTCACG	1353–1370	220	[35]
		PDVdpR	CCTTTAATGAGTCCGT	1572–1557		[35]
		PDVcpF	CTTCCAACTTTCGACTGTTTG	1143-1162	687	[37]
		PDVcpR	TCATCCACTGACTATTTTATCC	1830-1809		[37]
		PDVrna2F	TGATGACGTCGAAGAGCAAG	550-569	418	[37]
		PDVrna2R	GGGACAAGTCGAAAGAGCAG	949–968		[37]
General primers	pUPD2 plasmid	pUDP2-R2	GAGGAAGCCTGCATAACG	n.a.	Various	[38]
		pUPD2-F2	CCCGATCAACTCGAGTGCCA	n.a.		[38]

* nontemplate bases in intalics
